# Metagenomic compendium of 189,680 DNA viruses from the human gut microbiome

**DOI:** 10.1038/s41564-021-00928-6

**Published:** 2021-06-24

**Authors:** Stephen Nayfach, David Páez-Espino, Lee Call, Soo Jen Low, Hila Sberro, Natalia N. Ivanova, Amy D. Proal, Michael A. Fischbach, Ami S. Bhatt, Philip Hugenholtz, Nikos C. Kyrpides

**Affiliations:** 1 Environmental Genomics and Systems Biology Division, Lawrence Berkeley National Laboratory, Berkeley, CA USA; 2 U.S. Department of Energy Joint Genome Institute, Berkeley, CA USA; 3 Australian Centre for Ecogenomics, School of Chemistry and Molecular Biosciences, The University of Queensland, Brisbane, Queensland Australia; 4 Department of Medicine (Hematology), Stanford University, Stanford, CA USA; 5 Department of Genetics, Stanford University, Stanford, CA USA; 6 PolyBio Research Foundation, Kenmore, WA USA; 7 Department of Bioengineering, Stanford University, Stanford, CA USA; 8 Department of Microbiology and Immunology, Stanford University, Stanford, CA USA; 9 ChEM-H Institute, Stanford University, Stanford, CA USA; 10 Chan Zuckerberg Biohub, San Francisco, CA USA

**Keywords:** Bacteriophages, Data mining

## Abstract

Bacteriophages have important roles in the ecology of the human gut microbiome but are under-represented in reference databases. To address this problem, we assembled the Metagenomic Gut Virus catalogue that comprises 189,680 viral genomes from 11,810 publicly available human stool metagenomes. Over 75% of genomes represent double-stranded DNA phages that infect members of the Bacteroidia and Clostridia classes. Based on sequence clustering we identified 54,118 candidate viral species, 92% of which were not found in existing databases. The Metagenomic Gut Virus catalogue improves detection of viruses in stool metagenomes and accounts for nearly 40% of CRISPR spacers found in human gut Bacteria and Archaea. We also produced a catalogue of 459,375 viral protein clusters to explore the functional potential of the gut virome. This revealed tens of thousands of diversity-generating retroelements, which use error-prone reverse transcription to mutate target genes and may be involved in the molecular arms race between phages and their bacterial hosts.

## Abstract (web summary)

Almost 190,000 draft-quality DNA virus genomes are recovered by mining more than 11,000 deposited human stool metagenomes to improve resources for understanding the human gut virome.

## Main

The gut microbiome is a complex microbial ecosystem with important roles in human health and development^1^. Although often overlooked, viruses are estimated to be abundant in the microbiome^2,3^ and have been associated with human disease^4–6^. In particular, bacteriophages (viruses that infect bacteria) constitute the majority of viral particles^3,7,8^ and can impact microbial ecosystem processes through phage predation^9^, lysogeny^10^ and horizontal gene transfer^11^. Despite their ubiquity, our knowledge of viral genomic diversity in the microbiome is limited, with most viral sequences failing to match existing genome databases^8^. A comprehensive database of viral genomes from the microbiome is a prerequisite for assembly-free quantification of viruses, predicting host–virus interactions^12^, comparative genomics and genome mining (for example, anti-CRISPR genes^13^).

Traditionally, there have been two main approaches for sequencing viral genomes from the microbiome: viral metagenomic sequencing and bulk metagenomic sequencing. Viral metagenomics involves using size filtration to select for virus-like particles, followed by viral DNA extraction, (often) whole-genome amplification, shotgun sequencing and metagenomic assembly^14–17^. Although size filtration is used to enrich extracellular viruses, it will not remove all cellular organisms^18^ and can exclude some large viruses^19^. Whole-genome amplification is often necessary due to low sample biomass but can skew viral abundances and over-amplify small circular single-stranded DNA (ssDNA) viruses^19–21^.

An alternative approach is to generate bulk metagenomes, without size filtration or whole-genome amplification, followed by computational separation of viral and cellular sequences^22,23^. This approach captures sequences of both extracellular and intracellular viruses, including integrated prophages, and is not biased by whole-genome amplification. However, with bulk metagenomic sequencing, it is more challenging to assemble low-abundance viruses because the majority of reads derive from cellular organisms^24^. Additionally, DNA-extraction protocols may not be optimized for viruses^16^ and some viral sequences may originate from degraded prophages in bacterial chromosomes^10,25^.

To date, numerous studies have used viral metagenomic sequencing to identify phage genomes from human stool samples across a wide variety of phenotypes^4–6^. To integrate these disparate data sets, Soto-Perez et al.^26^ formed the Human Virome Database (HuVirDB) from 1,831 public samples (including skin, stool, lung and blood) and Gregory et al.^27^ formed the Gut Virome Database (GVD) from 2,697 public samples. In contrast to these viral metagenomic studies, Paez-Espino et al^22^. formed the IMG/VR database by identifying viruses from bulk metagenomes, including 490 stool samples from the Human Microbiome Project^28^. Since this publication, the number of publicly available bulk metagenomes has rapidly grown, as evidenced by recent, large-scale data mining efforts^29–31^.

To expand these existing resources and provide a complementary view of the gut virome, we performed large-scale identification of viral genomes from 11,810 bulk metagenomes from human stool samples derived from 61 previously published studies. We used these data to form the Metagenomic Gut Virus (MGV) catalogue, which contains 189,680 viral draft genomes estimated to be >50% complete and representing 54,118 candidate viral species. These genomes vastly expand the known diversity of DNA viruses from the gut microbiome and improve knowledge of host–virus connections. We expect the MGV catalogue will be a useful community resource for interrogating the role of the gut virome in human health and disease.

## Results

### A genomic catalogue of DNA viruses from the gut microbiome

**Fig. 1:**
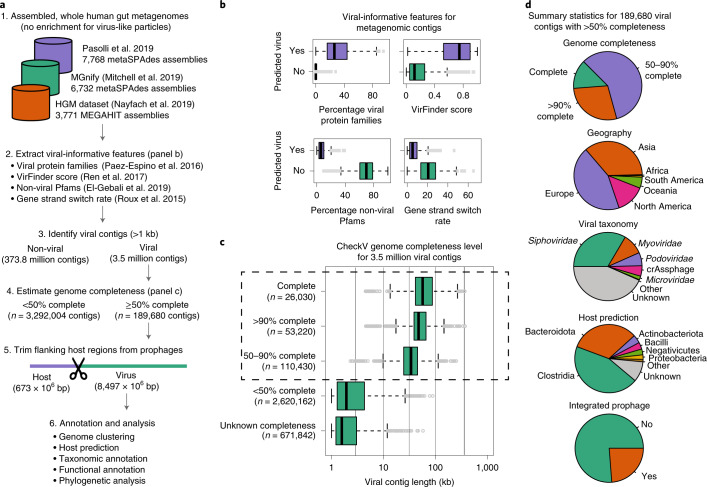
Thousands of high-quality viral genomes recovered from human gut metagenomes. **a**, Overview of viral discovery effort and formation of the MGV catalogue. **b**, Genomic signatures of predicted viral and non-viral metagenomic contigs longer than 20 kb. Displayed data is for 1,000 randomly selected contigs from each category. **c**, Distribution of estimated genome completeness and classification of MGVs into quality tiers (complete, *n* = 26,030; >90% complete, *n* = 53,220; 50–90% complete, *n* = 110,430; <50% complete, *n* = 2,620,162; completeness not determined, *n* = 671,842). **d**, Metadata and annotations for 189,680 genomes with >50% completeness. For box plots, the middle line denotes the median, the box denotes the interquartile range and the whiskers denote 1.5× the interquartile range.

We developed a viral detection pipeline for the current study using a combination of well-established methods and signatures, including VirFinder^32^, viral protein families from the Earth’s Virome Study^23^, and the propensity for viral genes to lie on the same strand^33^ and be functionally unannotated^8^ (Fig. 1a,b). Based on in silico benchmarking, our pipeline was able to sensitively identify genome fragments of diverse human-associated viruses and phages, including crAss-like phages^34^ and megaphages^35^, with high specificity and performed favourably compared with existing methods (Supplementary Tables 1–2 and Methods). For genome fragments of 1, 10 and 100 kb our pipeline achieved true-positive rates (TPR) of 41%, 74% and 96% at false-positive rates (FPR) of only 0.43%, 0.38% and 0.18%.

**Extended Data Fig. 1:**
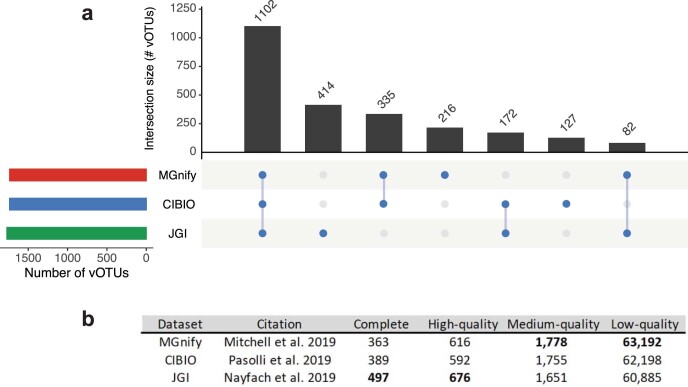
Impact of assembly methods on viral recovery from gut metagenomes. The MGV catalogue was formed using metagenomic viral contigs identified from three studies that performed large-scale assembly of human stool metagenomes. The CIBIO and MGnify studies used MetaSPAdes for metagenomic assembly while the JGI study used MEGAHIT. To explore the effect of assembler on virus identification, we compared viral contigs identified from a common set of 752 stool samples which were assembled by all three studies and were each represented by a single SRA run accession. **a**, The number of vOTUs represented by viral contigs (>50% completeness) from each of the three studies. A similar number of vOTUs were identified from metagenomic contigs assembled by each study. **b**, The number of viral contigs at different quality levels identified from each of the three studies. A greater number of complete and high-quality viral genomes are recovered from the MEGAHIT assemblies.

We then applied our pipeline to bulk metagenomes from 11,810 distinct human gut samples that were assembled in previous studies^29,31,36^ to broadly capture lytic and lysogenic DNA viruses (Fig. 1a and Supplementary Table 3). The analysed data sets span 61 studies across 24 countries and include individuals with a wide range of ages, lifestyles and disease states (Supplementary Table 4). This revealed 3.5 million unique, single-contig viral genomes longer than 1 kb. Based on an analysis of metagenomes found in all three studies, we found that choice of assembler (that is, MEGAHIT versus metaSPAdes) had little effect on the quality or identity of recovered viruses (Extended Data Fig. 1). Viral genomes were largely derived from individuals in Europe (46%), China (23%) and the USA (13%) reflecting the amount of metagenomic data from these sources (45%, 24% and 11% of total assembly length, respectively).

The completeness of metagenome-assembled viruses can vary widely, ranging from short fragments to complete or near-complete genomes. To assess genome completeness, we applied CheckV^37^, revealing 189,680 genomes that were at least 50% complete (Fig. 1c), including 26,030 complete genomes identified on the basis of direct terminal repeats (*n* = 19,704), host–provirus boundaries (*n* = 5,123) and inverted terminal repeats (*n* = 1,203). To improve genome quality, we removed flanking host regions from these sequences (Fig. 1a); confirming that viral genomes were free of host contamination, we identified only one full-length 16S rRNA gene (flanking an integrated prophage) among all 189,680 viruses compared with 83,050 16S rRNA genes in the full set of metagenomic contigs used for viral discovery (Methods). We focused all subsequent analysis on the 189,680 genomes with >50% completeness to avoid limitations associated with small genome fragments^38^ and to be consistent with quality standards applied to microbial genomes^39^.

Because there was no separation of viral-like particles prior to sequencing, we anticipated many viruses were derived from bacterial chromosomes. However, only 24% of viral genomes had evidence of host integration (Fig. 1d) and only 10% where the flanking host region was >5 kb. Furthermore, the majority of non-integrated viruses were classified as virulent based on BACPHLIP^40^ (65% of 140,689) which is a computational tool that predicts bacteriophage lifestyle from conserved protein domains. Likewise, BACPHLIP classified 58% of the 26,030 complete genomes as virulent, indicating that this result is not due to incomplete genome assembly because integrase genes often occur at the ends of prophage genomes^41^. Together, these results demonstrate that it is not uncommon to recover the genome sequences of lytic viruses from unfiltered stool metagenomes.

### Host prediction and taxonomic annotation

**Fig. 2:**
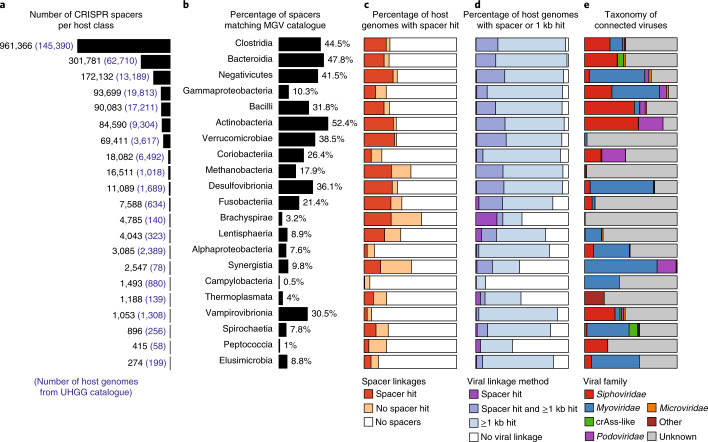
Viral connections to human gut Bacteria and Archaea. **a**, Bar plots indicating the number of CRISPR spacers across 286,997 human gut Bacteria and Archaea, with the number of genomes indicated in parentheses. Each row indicates one host class containing at least 20 genomes and 100 spacers. The majority of CRISPR spacers are derived from Clostridia and Bacteroidia, reflecting their abundance in the human gut. **b**, Percentage of CRISPR spacers matching viral genomes with a maximum of one mismatch. **c**, Host genomes containing a CRISPR-spacer array, and those with a CRISPR-spacer array match to a viral genome. **d**, Genomes linked to a virus using a combination of approaches as indicated. **e**, Distribution of known viral families that are associated with each host class. Each host class is infected by a distinct repertoire of viral families.

Predicting the cellular hosts of viruses is important for understanding phage predation and an essential first step towards utilizing host–virus interactions to design innovative phage therapies^42^. Towards this goal, we leveraged the Unified Human Gastrointestinal Genome (UHGG) database of 286,997 genomes of Bacteria and Archaea from the gut microbiome^43^, which represents 4,644 prokaryotic species (Fig. 2). First, we extracted 1,846,441 CRISPR spacers from the UHGG genomes, and looked for near-exact matches to the 189,680 viral genomes, resulting in host–virus connections that covered 81% of viruses (*n* = 153,892). Interestingly, just 21% of viruses were connected to a host when using spacers extracted from the 4,644 species-level representatives, indicating considerable CRISPR diversity between bacterial strains and active community infection. Although most viruses were targeted by a spacer, CRISPR arrays were found in only 28% (*n* = 79,734) of UHGG genomes and in <1% of many prevalent species including *Alistipes putredinis*, *Bacteroides cellulosilyticus* and *Bifidobacterium breve*, confirming the limited distribution of this anti-viral defence system^44^. To expand the host–virus network, we performed whole-genome alignment between the 189,680 viruses and 286,997 hosts and identified connections based on near-exact genomic matches (≥96% identity over ≥1 kb), resulting in connections that covered 96% of host genomes and 90% of viral genomes. As expected, the majority of viruses were connected to *Firmicutes* (predominantly Clostridia*)* and Bacteroidia, which are the two dominant phyla of bacteria in the gut microbiome (Fig. 1d). These results show that host–virus interactions can be systematically elucidated through extensive assembly of both viral and microbial genomes from the same environment.

Next, we assigned viruses to families from the ICTV database based on alignments to genomes from NCBI GenBank and crAss-like viruses from recent studies^34,45,46^ (Fig. 1d). Only 56.6% of viruses could be annotated at the family level, confirming a large knowledge gap in the taxonomy of human gut viruses^8^. To increase sensitivity, we used taxonomically informative profile hidden Markov models (HMMs) from the VOG database (http://vogdb.org), revealing most unannotated viruses to be members of the *Caudovirales* order. Among annotated sequences were 9,395 genomes of putative crAss-like viruses (5% of total). Overall, only 0.51% (*n* = 48) of the putative crAss phages displayed clear evidence of lysogeny (that is flanked by host region and contained an integrase), which was more than 17× lower than other viruses in the data set. Consistent with this, 56% of high-quality crAssphage genomes (*n* = 5,439) could be circularized compared with 24% of the other high-quality genomes (*n* = 36,872). crAss-like genomes contained several other unusual features, including low GC content (mean = 32%), usage of an alternative genetic code and a predominance of hypothetical proteins. For example, TAG or TGA stop codons were recoded to amino acids in 27% of crAss-like phages versus 0.5% of other viruses. Likewise, only 12% of crAssphage proteins had significant hits to Pfam, KEGG or TIGRFAM versus 28% of proteins from other viruses. This large-scale analysis supports previous findings that some crAss-like viruses have an obligate lytic lifestyle^46^ and reveals several unusual features that further establish crAssphage as an outlier among human gut viruses^47^.

### Vastly expanded viral genomic diversity

**Fig. 3:**
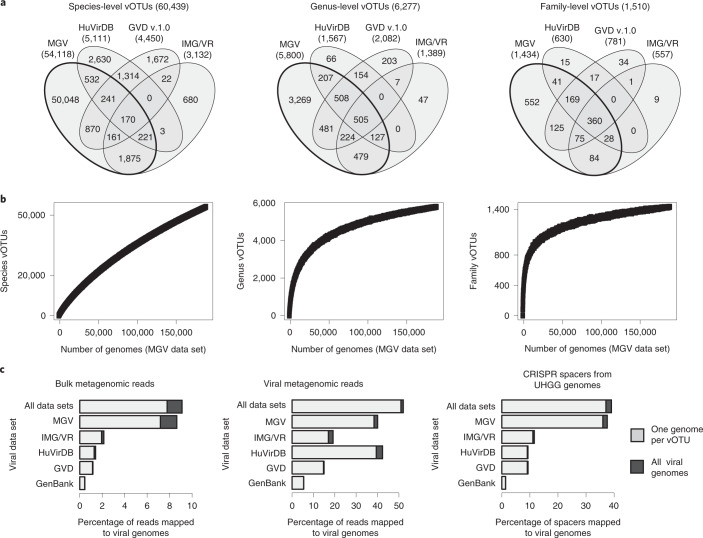
Genome clustering and comparison with existing databases. The 189,680 genomes from the MGV catalogue were compared with human gut virus genomes >50% complete from three databases: IMG/VR (*n* = 6,895), HuVirDB (*n* = 9,626) and GVD (*n* = 4,494). **a**, Viral genomes were clustered into vOTUs at approximately species, genus and family levels. **b**, Accumulation curves for vOTUs from the MGV catalogue. **c**, Percentage of reads from 1,257 unfiltered stool metagenomes, percentage of reads from 585 viral stool metagenomes and percentage of CRISPR spacers from 286,997 UHGG genomes mapped to viral genomes from various databases.

To quantify the diversity of genomes in the MGV catalogue, we first identified species-level viral operational taxonomic units (vOTUs) using the MIUViG recommended criteria of 95% average nucleotide identity (ANI) over 85% of the length of the shorter sequence^38^. Small adjustments to these parameters did impact the number of identified vOTUs, suggesting a continuum of viral diversity beyond the species-level boundary (Supplementary Table 5). Overall, we identified 54,118 vOTUs, of which 8,086 included members from at least two samples (Fig. 3a). The largest vOTUs were predicted to infect some of the most prevalent species in the gut microbiome, including *Bacteroides uniformis*, *Faecalibacterium prausnitzii* and *Agathobacter rectalis* (formerly *Eubacterium rectale*). To identify higher-ranking viral clades, we clustered genomes into approximately genus- and family-level groups on the basis of pairwise average amino acid identity (AAI) and gene sharing (Methods), revealing 5,800 genus-level vOTUs and 1,434 family-level vOTUs (Fig. 3a). Accumulation curves of vOTUs appeared to be approaching an asymptote at the family and genus ranks but not yet for species (Fig. 3b).

Other recent studies have also compiled databases of DNA viruses from the gut microbiome^22,26,27^. To identify vOTUs unique to the MGV catalogue, we clustered the 189,680 genomes from our study together with medium- and high-quality viral genomes from three other genome catalogues (Fig. 3a): the HuVirDB (9,626 genomes derived from 1,543 viral metagenomes), GVD v.1.0 (4,494 genomes derived from 471 viral metagenomes and 98 whole metagenomes) and IMG/VR v.2.0 (6,895 genomes derived from 490 whole metagenomes). Note that during the review of this manuscript, the IMG/VR and GVD were updated to new versions which were not analysed here. To enable comparability between all studies, CheckV was run on all viral data sets and genome fragments with <50% completeness were excluded.

Strikingly, we found that 50,048 of the 54,118 species-level vOTUs from the MGV catalogue (92%), comprising 100,398 of the 189,680 genomes (53%), did not cluster with any genome from the other databases (Fig. 3a). In contrast, the three reference databases combined represented 10,391 species-level vOTUs, nearly half of which were also found in the MGV. The MGV and IMG/VR databases, which were both derived from whole metagenomes, shared the greatest number of vOTUs and contained a relatively high proportion of lysogenic phages from the order *Caudovirales*, whereas the HuVirDB and GVD data sets, which were largely derived from viral metagenomes, were enriched in small circular ssDNA viruses from the *Microviridae*, *Anelloviridae* and *CRESS* families.

Next, we compared the four genome catalogues based on their ability to recruit sequencing reads from a geographically diverse set of whole metagenomes and viral metagenomes (Fig. 3c). To prevent self matches we discarded alignments between sequencing reads and viral genomes derived from the same original study. Overall, MGV genomes recruited 8.6% of whole-metagenome reads, which was 4.0-fold higher than any other database, and 40.1% of virome reads, which was comparable with the HuVirDB at 42.3%. We also compared the recruitment of CRISPR spacers to each viral database as a way of quantifying host–virus connections (Fig. 3c). Overall, 37.5% of the 1.8 M spacers from UHGG genomes matched a genome from the MGV catalogue, which was 3.25-fold higher than any other database. The number of matched spacers and metagenomic reads did not change considerably when using a viral database of only species-level representatives (Fig. 3c). Together, these results show that the MGV catalogue has substantially increased known viral diversity, improved detection of viral reads in whole metagenomes and expanded coverage of host–virus connections.

### Phylogenomics of intestinal *Caudovirales*

**Fig. 4:**
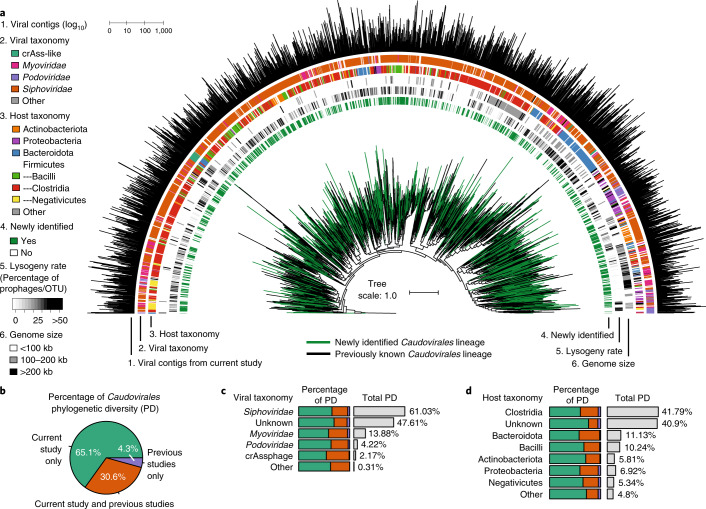
Phylogenomics of intestinal *Caudovirales*. A phylogenetic tree was constructed from 25,528 species-level genomes derived from the MGV and other databases (IMG/VR, HuVirDB and GVD). **a**, Phylogeny of intestinal *Caudovirales*. Tree was plotted using iToL^74^ and to improve visualization only one genome per genus-level vOTU is displayed. Branch colour indicates whether a lineage is represented by a previously published study (black) or is unique to the MGV catalogue (green). Outer rings display metadata for each vOTU. **b**, PD was calculated by taking the sum of branch lengths represented by species-level viral genomes. **c**,**d**, MGVs from the current study result in a large gain in PD, which is consistent across (**c**) viral families and (**d**) viruses infecting different host groups.

*Caudovirales* comprise an expansive order of tailed double-stranded DNA (dsDNA) phages found in numerous environments^48^ and were highly represented in the stool metagenomes we analysed. To explore the evolution of this group in the gut microbiome, we constructed a species-level phylogenetic tree based on a concatenated alignment of 77 protein-coding marker genes (Fig. 4a)^49^. After removing genomes with insufficient data (fewer than three markers or <5% representation in alignment), the final tree contained 25,528 species-level viral genomes derived from the four databases of uncultivated gut viruses (MGV, IMG/VR, HuVirDB and GVD).

Based on cumulative branch length, the MGV catalogue covered 95.7% of the total phylogenetic diversity (PD) and contained genomes representing all major lineages across the tree (Fig. 4b). Compared with the three other databases combined, MGV genomes resulted in a 287% increase in PD that was evenly distributed across viral and host taxonomic groups. Clostridia phages were by far the most diverse group (41.8% of PD) because of the large number and broad phylogenetic distribution of these vOTUs. In contrast, *Bacteroidota* phages represented only 11.1% of PD with most vOTUs falling into four primary clusters (Fig. 4a) including one dominated by crAss-like phages (2.17% of PD). Overall, there was poor correspondence between the classical viral families based on tail morphology and genome-based phylogeny (for example, nearly all lineages contained *Siphoviridae* annotated genomes) which further highlights the need for a phylogeny driven taxonomy of *Caudovirales*^49^ and other viral groups, analogous to the GTDB taxonomy developed for Bacteria and Archaea^50^.

**Extended Data Fig. 2:**
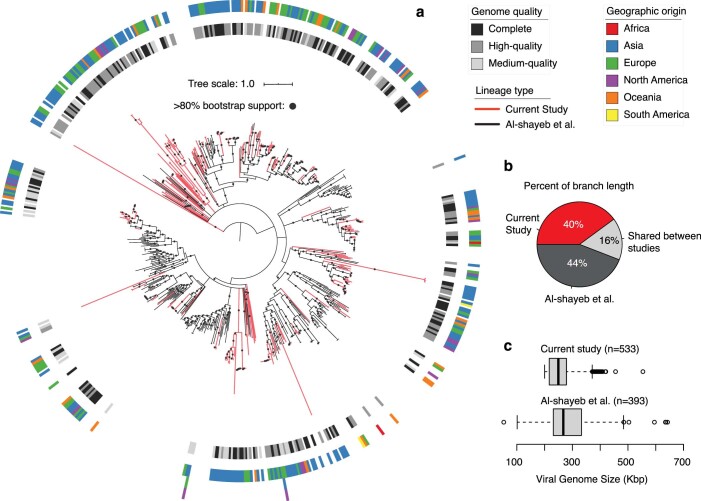
Diversity of jumbo phages identified in the MGV dataset. The tree includes MGV sequences alongside a reference set of metagenome-assembled jumbo phages published by Al-Shayeb et al.^51^. Branches leading to MGV sequences, or clades composed exclusively of MGV sequences, are highlighted in red. Nodes with support < 50% were collapsed, and nodes with support ≥ 80% are indicated with a grey circle on the corresponding branch. Outer rings indicate the genome quality and continent of origin for MGV sequences. When sequences from different continents were 100% identical and only 1 sequence was included in the tree, the different continents of origin are indicated with stacked coloured squares. For box plots, the middle line denotes the median, the box denotes the interquartile range (IQR), and the whiskers denote 1.5× the IQR.

Notably, several lineages contained jumbo phages with genomes exceeding 200 kb (518 genomes from 245 species-level vOTUs). As with other analyses, we carefully removed flanking host regions as well as assembly artefacts resulting in the same genome being repeated multiple times (Methods). The largest genome was a 553,716-bp near-complete linear genome closely related to a *Prevotella* phage Lak-A1 (ref. ^35^; 94.5% AAI over 87.1% of genes). As with crAss-like phages, jumbo phages were rarely integrated into a host (*n* = 13) although they sometimes contained integrases (*n* = 121). To characterize the diversity of these viruses in greater detail, we constructed a separate tree based on the large terminase subunit (TerL). Compared with a recently published collection of jumbo phages from diverse environments^51^, MGVs resulted in a large expansion of phylogenetic diversity and coverage of most lineages (Extended Data Fig. 2).

**Extended Data Fig. 3:**
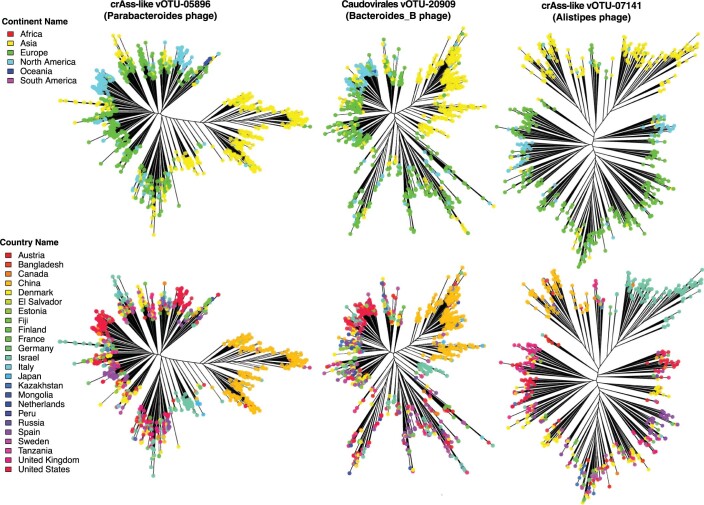
Strain level phylogeography of prevalent human gut phages. Core-genome SNP phylogenies were constructed for individual species-level vOTUs with at least 100 genomes. The figure shows three distinct vOTUs displaying a strong signature of phylogeography. For each tree, viral genomes are displayed as tips with colours indicating the geographic origin of the metagenomic sample.

Interestingly, jumbo phages and other *Caudovirales* appeared to have little to no preference in biogeographic distribution, as most clades were found in all continents. We hypothesized that region-specific phylotypes might be apparent over shorter evolutionary timescales, as observed for human gut bacteria^52^. Towards this goal, we used single-nucleotide variants to construct strain-level phylogenies for 146 prevalent vOTUs with more than 100 members (Methods). Strikingly, we observed discrete subspecies that were highly enriched in specific geographic regions for many vOTUs (Extended Data Fig. 3). For example, one crAss-like subspecies predicted to infect *Parabacteroides* was prevalent among samples from Asia, but rare or absent from Europe and North America. More work is needed to understand the evolutionary drivers and genomic adaptations underlying these phylogenetic patterns.

### Functional capacity of the gut virome

**Fig. 5:**
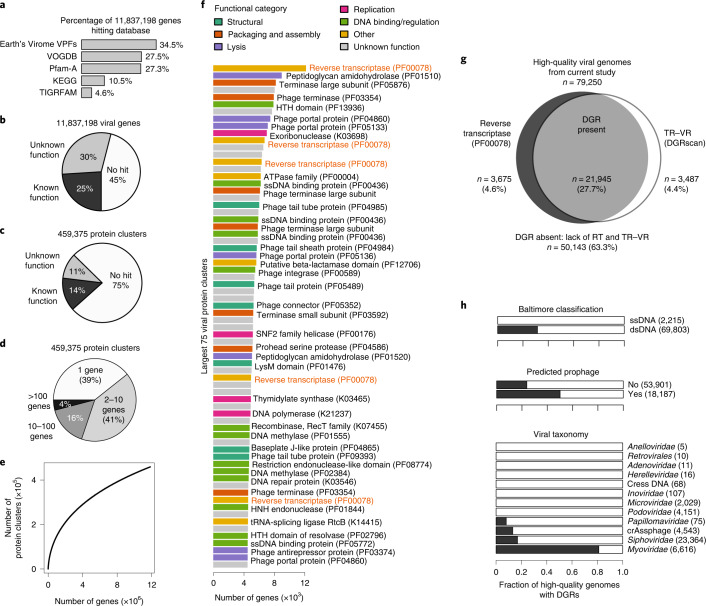
Functional landscape of intestinal phages. **a**, Protein-coding viral genes were identified across all MGVs and compared with profile HMMs from five databases. **b**, Forty-five per cent of genes fail to match any HMM, 30% match an HMM of unknown function and 25% match an HMM of known function. **c**, The 11,837,198 genes were clustered at 30% AAI using MMseqs2 into 459,375 protein clusters. **d**, Size distribution of protein clusters. **e**, An accumulation curve of protein clusters has not reached an asymptote. **f**, Functional annotations for the largest 75 protein clusters. Reverse transcriptases are highlighted in red. **g**, Prediction of DGRs based on the combination of the reverse transcriptase gene (PF00078) and TR–VR pair identified using DGRscan. A large fraction of MGVs contain the DGR system. **h**, DGR prevalence across different categories of viruses. DGRs are most common in lysogenic, dsDNA viruses from the *Myoviridae* family.

Although the functional potential of human gut bacteria and archaea has been extensively studied^43,53,54^, that of intestinal phages is less well understood. To explore this, we identified 11,837,198 protein-coding genes with at least 20 amino acids (98.4% with start and stop codons) across the 189,680 viral genomes from our study and compared these with HMM databases, including KEGG^55^, TIGRFAM^56^, Pfam^57^, VOGDB (http://vogdb.org/) and the Earth’s Virome database^23^. Overall, 45% of viral genes did not have significant matches to any database and 75% were not assigned any biological function (Fig. 5a,b), indicating that remarkably little is known about the functional potential of human gut viruses.

To identify the most common functions among intestinal phages, we clustered the 11.8 million viral genes at 30% AAI using MMseqs2 (ref. ^58^) into 459,375 de novo viral protein clusters (Fig. 5c) including 61% with at least two members (Fig. 5d). An accumulation curve displayed no plateau, indicating that gut phages have a large reservoir of functional diversity that is not fully captured by this study (Fig. 5e). Clostridia phages contained the most functional diversity with 173,187 protein clusters, reflecting the large phylogenetic diversity of these phages. Several of the largest protein clusters had no predicted function, including the fourth largest with 8,319 genes, and are therefore good candidates for experimental characterization in the future (Fig. 5f). Other large clusters were annotated with typical viral functions, including capsid formation, packaging, lysis, lysogeny, replication and transcriptional regulation (Fig. 5f).

**Extended Data Fig. 4:**
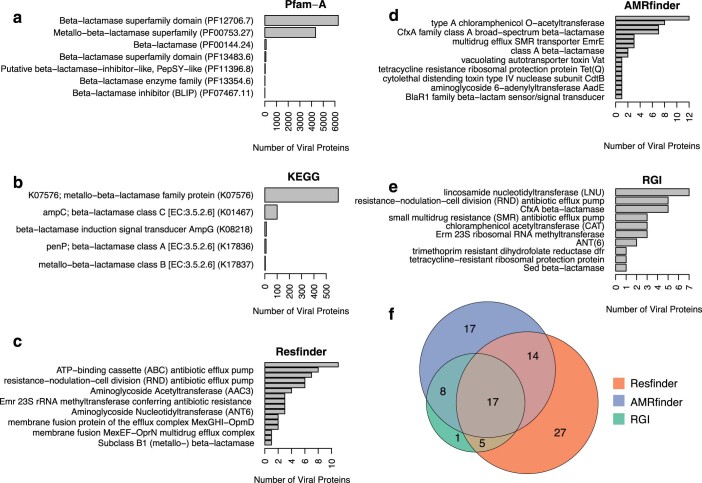
Antibiotic resistance genes identified from 11.8 million viral proteins. **a-b**, Viral genes with putative beta-lactamase domains identified based on hits to the Pfam and KEGG databases, respectively. **c-e**, Resistance genes (including beta-lactamases) identified using Resfinder, AMRfinder, or the Resistance Gene Identifier (RGI), respectively. **f**, Overlap of resistance genes identified by Resfinder, AMRfinder, and RGI. Most viral proteins identified with putative beta-lactamase domains are not confirmed as antibiotic resistance genes.

Although it is outside the scope of this article to enumerate all viral functions and auxiliary metabolic genes, we explored two particularly unusual findings. Based on HMM searches against Pfam, we uncovered 11,496 putative viral beta-lactamases (PF12706), including the majority of sequences in a single protein cluster with 5,832 members (Fig. 5f). Beta-lactamases are enzymes that enable resistance to beta-lactam antibiotics such as penicillins, cephalosporins and cephamycins, and pose a major global health problem^59^. To validate this result, we performed homology searches against curated databases of antimicrobial resistance genes using Resfams^60^, the NCBI AMRFinder^61^ and the Resistance Gene Identifier (RGI)^62^. These tools revealed a combined total of only 88 resistance genes (63 using Resfams, 56 using AMRFinder and 30 using RGI), indicating low similarity between the 11,496 putative viral beta-lactamases and validated resistance genes (Extended Data Fig. 4). Although functional metagenomic assays may uncover bona fide viral beta-lactamases in the gut microbiome, these results appear to support the conclusion that phages rarely encode antibiotic resistance genes^63^.

Another interesting finding was a large number of phage reverse transcriptases (RTs) (Fig. 5f and Supplementary Table 6). Overall, the RT domain (PF00078) was the third most common functional annotation, next to only the helix–turn–helix DNA-binding domain (PF01381) and phage integrase family (PF00589). RTs are known to occur in retroviruses^64^, RNA-targeting CRISPR–Cas systems^65^ and diversity-generating retroelements (DGRs)^66^. DGRs utilize error-prone reverse transcription to generate random mutations in the transcript of a template region (TR), which is then inserted back into the genome at a variable region (VR), thereby generating population-level hyper-variability in a specific gene. Since the DGR system was first characterized in a *Bordetella* bacteriophage^66^, it has been found in human microbiomes^67^ and in several human gut phages^68,69^.

To determine whether the viral RTs were part of the DGR system, we used the tool DGRscan^67^ to identify TR–VR pairs across 79,250 high-quality viral genomes with >90% estimated completeness. Confirming our hypothesis, the great majority of genomes with an RT also contained a TR–VR (85.7% of 25,620) compared with a small minority of those without an RT (6.5% of 53,630) (Fig. 5g). DGRs were remarkably common in certain *Caudovirales* families (for example, 84% of 6,616 *Myoviridae*) and among lysogenized viruses (50.1% of 18,187), whereas they were rare or completely absent from other *Caudovirales* families, ssDNA viruses and eukaryotic viruses (Fig. 5h). Although the vast majority of DGR gene targets were not functionally annotated, we observed highly significant enrichment within several Pfam domains (Supplementary Table 7) including an immunoglobulin-like domain that was 5.9-fold more common among DGR-targeted genes and is believed to play a role in phage interactions with carbohydrates on the cell surface of bacteria^70^. Together, these results reveal DGRs to be more common in intestinal phages than previously thought and may point towards viral proteins involved in molecular phage–host interactions.

## Discussion

In this study, we performed large-scale data mining of publicly available metagenomes to identify 189,680 draft-quality viral genomes representing an estimated 54,118 species-, 5,800 genus- and 1,434 family-level vOTUs. This large resource contains extensive viral genomic diversity not found in other databases, improves detection of viral reads in microbiomes and represents numerous diverse and previously uncharacterized viral groups. Through a combination of approaches, we were able to predict host–virus linkages that cover the majority of viral and prokaryotic diversity in the gut microbiome. These host–virus linkages may be important in the future for understanding disease processes, designing phage therapies or understanding host–virus co-evolutionary dynamics. Despite large-scale annotation efforts, we were only able to assign preliminary biological functions to 25% of viral genes, indicating that more work and new methods are needed to predict protein function in viral genomes, such as deep learning^71^ and functional metagenomic assays^72^. Although the current study focused exclusively on DNA viruses, future studies could use metatranscriptomics data to study RNA viruses or gene expression patterns.

**Extended Data Fig. 5:**
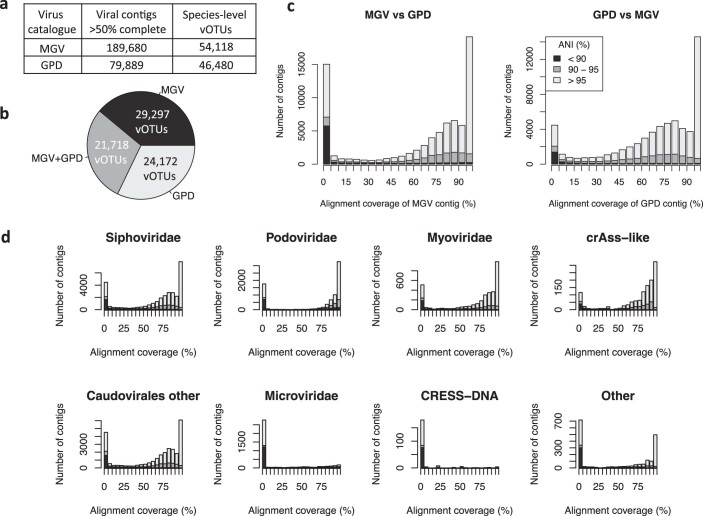
Comparison of viral contigs from the MGV and GPD catalogues. **a**, The number of viral contigs with at least 50% completeness from the MGV and GPD catalogues. The GPD catalogue contains 142,809 viral contigs when including those with <50% completeness. Contigs from each catalogue where clustered at 95% ANI over 85% the length of the shorter sequence to form species-level vOTUs. **b**, MGV and GPD catalogues were clustered together using the longest contig from each vOTU. **c**, The histograms show the similarity between contigs from the MGV (n = 54,118) and GPD (n = 46,480) catalogues. **d**, Similarity to the GPD catalogue for MGV contigs from different viral families: Siphoviridae (n = 22,513), Podoviridae (n = 5,075), Myoviridae (n = 2,560), crAss-like (n = 948), Caudovirales other (n = 19,633), Microviridae (n = 2,133), CRESS DNA (n = 115), other (n = 1,141).

While this manuscript was in review, Camarillo-Guerrero et al.^73^ published the Gut Phage Database (GPD), a collection of ∼142,000 non-redundant viral genomes (>10 kb) identified from 28,060 human gut metagenomes and 2,898 gut bacterial genomes. After applying CheckV, we found the GPD represents 79,889 viral contigs with >50% completeness that form 46,480 species-level vOTUs, which is 14% less than the 54,118 vOTUs from the MGV (Extended Data Fig. 5). Differences between viral catalogues are due to several factors, including data sets used for metagenome mining, methods for viral identification and criteria for sequence inclusion. For example, the MGV had greatly improved coverage of *Microviridae* which were excluded from the GPD due to their short length (mean = 4.9 kb). Combined, the MGV and GPD represented 75,187 species-level vOTUs, indicating that the two catalogues contain complementary viral diversity. In the future, these and other large-scale viral genome catalogues could be integrated to create a unified and standardized community resource, as recently performed for human gut microbial genome catalogues^43^.

## Methods

### Development of viral detection pipeline

We used a combination of four viral signatures to identify viral metagenomic contigs: (1) the presence of viral protein families, (2) the absence of microbial protein families, (3) the presence of viral nucleotide signatures, and (4) multiple adjacent genes on the same strand. For the presence of viral protein families, we used HMMs for 23,841 viral protein families from the IMG/VR database^23^ (downloaded 1 June 2019) after excluding 1,440 commonly found in microbial genomes or plasmids. For the absence of microbial protein families, we used HMMs for 16,260 protein families from the Pfam-A database^57^ (release 31) after excluding 452 commonly found in viruses. Proteins from metagenomic contigs were searched against HMMs from IMG/VR and Pfam-A using hmmsearch within the HMMER package v.3.1b2 (options: −Z 1, e-value: <1 × 10^−10^)^75^ and were classified as either viral or microbial based on the database containing the top hit. For the presence of viral nucleotide signatures, we applied the tool VirFinder v.1.1 (ref. ^32^) to metagenomic contigs, which scores sequences using a combination of k-mer frequencies and machine learning. For multiple adjacent genes on the same strand, we quantified the strand switch rate by dividing the number of strand switches by the number of genes on each contig.

### Benchmarking viral detection pipeline

We evaluated our viral detection pipeline on mock data sets we created that contained genome fragments from human-associated viruses and bacteria. Each mock data set contained genome fragments from six diverse categories of viruses: (1) crAss-like phages from the human gut^45^, (2) Lak-phages from human and mammalian microbiomes^35^, (3) bacteriophages assembled from human gut viromes^76^, (4) phages with CRISPR-spacer matches to gut isolated microbial genomes, (5) isolate dsDNA human viruses and (6) isolate ssDNA human viruses. Non-viral genome fragments were derived from: (1) gut isolated microbial genomes and (2) plasmids genomes. We generated 2,000 genomic fragments from randomly sampled genomes within each of the eight categories at each of seven different fragments lengths (1, 2, 5, 10, 20, 50 and 100 kb). The TPR (percentage of viral contigs classified as viral) and FPR (percentage of non-viral contigs classified as viral) were calculated for over 77,000 combinations of cut-off values for the four viral signatures. We selected up to five different combinations of cut-offs that resulted in the highest classification score for each fragment length, where the classification score was based on a weighted combination of the TPR and FPR (score = TPR − 50 × FPR; Supplementary Table 3). We assigned a very high negative weight to the FPR to avoid many false positives in the metagenomes which are expected to contain mostly non-viral sequences. We compared the performance of our method with VirSorter v.1.0.5 (ref. ^33^) and to VirFinder v.1.1 (ref. ^32^) using the same benchmark data set (Supplementary Table 2). VirFinder was run using default options and we applied *p*-value thresholds of 0.05, 0.01 and 0.001 for classifying genome fragments as viral. VirSorter was run with and without the ‘-virome’ option, and we used VirSorter categories 1 and 2 to classify a fragment as viral (excluding low confidence predictions and integrated prophages). We also evaluated VirSorter when including predicted prophages (categories 4 and 5).

### Application of pipeline to identify human gut viruses from whole metagenomes

To perform a comprehensive search for human gut viruses, we downloaded 18,271 publicly available metagenomic assemblies from human stool samples totalling 2.25 ×1012 bases and corresponding to 11,810 unique biological samples (Supplementary Table 1). Assemblies were obtained from two recent studies^29,31^ and the MGnify database (accessed on 16 April 2019)^36^. We excluded assemblies from environments other than human gut and those that could not be assigned to an accession number from the NCBI SRA database. Metadata were obtained from previous studies and the NCBI BioSample database^77^ (Supplementary Table 2). We applied our viral detection pipeline method to identify 4,436,008 contigs longer than 1 kb across the 18,271 metagenomic assemblies (Supplementary Table 1), which were de-replicated to 3,481,684 sequences at 100% ANI over 100% the length of the shorter sequence.

### Gene calling and identifying viruses with alternative genetic codes

Prodigal v.2.6.3 (ref. ^78^) was used to identify protein-coding genes in the 3,481,684 viral genomes using the flag ‘-p meta’ optimized for metagenomes. Additionally, we ran a custom pipeline to identify viruses using an alternative genetic code. Specifically, Prodigal was run using the standard code (11), and three alternative genetic codes: TGA recoded (code 4 or 25), TAG recoded (code 15) and TAA recoded (code 90), as previously described by Ivanova et al.^79^. To reduce false positives this procedure was only run on viral contigs longer than 10 kb with GC content <50%. For each viral contig, Prodigal outputs a GFF file that includes a coding potential score for every predicted gene. To evaluate the genetic codes, we took the sum of coding potential scores per contig. An alternative genetic code was predicted if it’s total coding potential score was the greatest and at least 10% greater than the standard genetic code.

### Viral reference genomes used for comparison

Viral genomes from the MGV were compared against four reference databases: IMG/VR v.2.0 (ref. ^22^), GVD v.1.0 (ref. ^27^), HuVirDB v.1.0 (ref. ^26^) and NCBI GenBank. For IMG/VR, we extracted 28,697 viral contigs which were identified from 490 whole metagenomes from human stool samples using the Earth’s Virome Pipeline^23^. For GVD, we used all 13,203 viral contigs, which were identified from 471 viral metagenomes and 98 whole metagenomes using a combination of tools including VirSorter and VirFinder and previously clustered into viral populations. An updated version of the GVD was released while the paper was under review but was not analysed here. For the HuVirDB, we extracted 929,886 contigs longer than 1 kb from 1,543 viral metagenomes from human stool samples. Because no viral prediction was previously applied, we ran the viral prediction pipeline developed for the current manuscript. For NCBI GenBank (downloaded 1 June 2019), we extracted 28,996 complete viral genomes after removing those labelled as incomplete, contaminated, or chimeric.

### Quality control of viral genomes

We applied CheckV v.0.7.0 (database v.0.6)^37^ to all viral sequences to identify closed genomes, estimate genome completeness and remove flanking host regions on assembled proviruses. Putative complete genomes were predicted based on direct terminal repeats (minimum 20 bp), inverted terminal repeats (minimum 20 bp) or provirus integration sites (host region predicted on both ends of viral contig), and were additionally required to display >90% estimated completeness based on comparison with CheckV reference genomes. A small number of sequences were removed that contained large repeats spanning >30% of the contig length. We selected all genomes with >50% estimated completeness for further analysis, resulting in 189,680 viral contigs from the MGV catalogue, 6,895 contigs from IMG/VR, 4,494 from GVD, 9,626 from HuVirDB and 28,996 from GenBank. We estimated the amount of non-viral DNA from cellular organisms among MGV sequences by searching for 16S and 18S rRNA genes using Barrnap v.0.9-dev (https://github.com/tseemann/barrnap) with models for Bacteria, Archaea and Eukaryotes. Alignments were required to cover ≥70% of the 16S or 18S rRNA gene and display an e-value <1 × 10^−5^. This same procedure was applied to the 18,271 metagenomic assemblies used for viral discovery to estimate the background levels of 16S and 18S rRNA genes.

### Taxonomic annotation

Viral genomes were annotated based on amino acid alignments to a database of proteins derived from complete NCBI GenBank genomes and crAss-like genomes. Annotations were performed using the Baltimore classification (DNA, dsDNA, ssDNA, ssDNA-RT, dsRNA, RNA, ssRNA, ssRNA-RT) as well the ICTV taxonomy at the order, family and genus ranks. DIAMOND v.0.9.32 (options: –query-cover 50–subject-cover 50–e-value 1e-5–max-target-seqs 1000)^80^ was used to align viral proteins to the reference database. The taxonomy of the top database hit was then transferred to each protein at each taxonomic rank (Baltimore, order, family, genus). In cases where the taxonomy of the top hit was missing, we used the next hit if its bit-score was within 25% of the top hit. For each viral genome, we aggregated annotations across proteins after weighting by bit-scores. Each viral genome was then annotated at the lowest taxonomic rank having >70% agreement across annotated proteins. At the family rank, we required genomes to have a minimum of two annotated proteins with >30% AAI to the database. At the genus rank, we required genomes to have a minimum of three annotated proteins with >40% average AAI to the database. As validation, we applied our pipeline to taxonomically annotated genomes from NCBI GenBank after removing closely related genes from the database. Our pipeline achieved average TPRs of 90.0%, 98.7%, 92.2% and 73.5% at precision values of 95.6%, 99.9%, 99.3% and 96.5% for taxonomic ranks of Baltimore, order, family and genus, respectively.

### Host prediction

We used a combination of CRISPR-spacer matches and ≥1 kb genome sequence matches to associate viral genomes to Bacterial and Archaeal genomes from the UHGG collection^43^. The UHGG contains 286,997 genomes, representing 4,644 species of Bacteria and Archaea from the human gut that are taxonomically annotated using GTDB-tk v.0.3.1 (GTDB release 89)^81^. Many of the UHGG genomes are metagenome-assembled genomes, which sometimes contain erroneously binned sequences, including those from viruses. To address this, we conservatively identified and removed 2,043,531 contigs from UHGG genomes where the host region comprised <50% of the contig length. We then compared the remaining UHGG contigs with viral genomes and identified ≥1 kb genome sequence matches with ≥96% DNA identity using blastn from the blast+ package v.2.9.0 (ref. ^82^). Next, we identified 1,846,441 spacers from 145,053 CRISPR arrays from 79,735 UHGG genomes using a combination of CRT^83^ and PILER-CR^84^ with default parameters. Redundant CRISPR arrays predicted by both tools were merged based on genomic coordinates. Spacers were searched against viral genomes using blastn from the blast+ package v.2.9.0 (options: -dust = no -word-size = 18), allowing a maximum of one mismatch or gap over ≥95% of the spacer length. For each viral genome, we then aggregated connections to UHGG genomes and identified the lowest host taxonomic rank resulting in >70% agreement across connections.

### Clustering viral genomes into vOTUs

All viral genomes with >50% completeness were clustered into species-level vOTUs on the basis of 95% ANI and 85% alignment fraction (AF) of the shorter sequence, as recommended by Roux et al.^38^. ANI and AF were estimated between all genome pairs using a custom script from the CheckV repository. The script performs all-versus-all local alignments using blastn from the blast+ package v.2.9.0 (options: perc_identity = 90 max_target_seqs = 10000). ANI is computed as the length-weighted average DNA identity across local alignments between each genome pair. AF is computed by merging alignment coordinates between each genome pair and dividing by the length of each genome. This approach gave consistent results compared to MUMMer4 (ref. ^85^), while running in a small fraction of the time. Clustering was performed using a greedy, centroid-based algorithm in which: (1) genomes were sorted by length, (2) the longest genome was designated as the centroid of a new cluster, (3) all genomes within 95% ANI and 85% AF were assigned to that cluster, and steps 2 and 3 were repeated until all genomes had been assigned to a cluster.

To identify genus- and family-level vOTUs, we clustered viral genomes using a combination of gene sharing and AAI. For computational efficiency, only the longest genome per species-level vOTU was included. Blastp from the DIAMOND package v.0.9.25.126 was used with options ‘-e-value 1 × 10^−5^–max-target-seqs 10,000’ to align all viral proteins. For each pair of genomes, we identified shared genes (e-value <1 × 10^−5^), computed their AAI, and computed the percentage of genes shared. Edges between genomes were filtered based on their minimum AAI and gene sharing. Clustering was performed with MCL v.14-137 using different values for the inflation factor parameter. We then selected the filtering thresholds and MCL inflation factor that resulted in the highest agreement with genus- and family-level annotations from NCBI RefSeq, respectively. At the family level, we filtered connections between genomes with <20% AAI or <10% genes shared and used an inflation factor of 1.2. At the genus level, we filtered connections between genomes with <50% AAI or <20% gene sharing and used an inflation factor of 2.0. We benchmarked our approach on taxonomically annotated genomes from NCBI, showing that viral clusters displayed high taxonomic homogeneity (that is the percentage of genomes from each cluster assigned to the same taxon; genus rank = 95.1%, family rank = 93.7%), though sometimes split known taxa into multiple clusters (that is percentage of genomes from each taxon assigned to the same cluster: genus rank = 92.6%, family rank = 74.5%).

### Metagenomic read recruitment

Read mapping was performed to viral genomes databases to assess their coverage of viruses in microbiomes. First, we downloaded short reads from human gut viromes analysed by the HuVirDB plus short reads from three recent gut virome studies^14,86,87^. Short reads from whole metagenomes were downloaded for 1,257 stool samples from various countries (representing up to 50 samples per country). To ensure that viromes were mostly free of cellular contamination, we ran the viromeQC tool^88^ and retained viromes with an enrichment score >10, as recommended by the authors. For computational efficiency, we only analysed the first 1,000,000 sequencing reads from each data set. For quality control, we discarded reads that were either too short (<70 bp), contained ambiguous base calls, had low base quality scores (mean quality score <30) or mapped to the human genome (build hg19).

Next, we used Bowtie v.2.3.2 (ref. ^89^) to construct genome indexes for read mapping. Five indexes were created using all genomes from each of the four gut human virus databases (MGV, IMG/VR, HuVirDB, GVD), plus NCBI GenBank. Five additional indexes were created using only a single genome per species-level vOTU. Next, we used Bowtie 2 (options ‘–very-sensitive -k 20’) to align sequencing reads to each of the 10 genome indexes. Alignments between sequencing reads and viral genomes derived from the same SRA study were discarded to prevent overestimation of mapping rates. Additionally, alignments with mapping identity <95% (for example, edit distance >5 for 100-bp read) were discarded. After these filtering steps we quantified the percentage of high-quality, non-human reads that mapped to each database.

### Phylogenetic analyses

We constructed a phylogeny of *Caudovirales* genomes using the method described by Low et al.^49^. First, we identified the set of 77 *Caudovirales* markers in the representative genomes of 60,439 species-level vOTUs. HMMs for the 77 markers were searched against the protein sequences and the top hits individually aligned to the profile HMMs using HMMER v.3.1b2. Individual marker alignments were then trimmed to retain positions with less than 50% gaps using trimAl v.1.4 (ref. ^90^) and concatenated, filling in gaps for missing markers where necessary. Only genomes containing at least three markers and having data at >5% of alignment columns were retained. This resulted in a multiple sequence alignment of 28,780 genomes with 22,711 alignment columns. We then inferred a concatenated protein phylogeny from the multiple sequence alignment using FastTree v.2.1.9 (ref. ^91^) under the WAG + G model with the additional flags ‘-mlacc 2’ and ‘-slownni’. The tree was then midpoint-rooted and visualized using iToL^74^.

In addition, we constructed core-genome single-nucleotide polymorphism (SNP) phylogenies of individual species-level vOTUs with at least 100 genomes. SNPs were identified by aligning all genomes to the longest genome in the cluster using nucmer from the MUMmer4 package v.4.0.0beta2 (ref. ^85^) with default options. SNPs were identified at genomic positions covered by ≥50% of genomes and we retained all genomes with data at ≥50% of positions. FastTree v.2.1.9 was used to construct phylogenetic trees using default options.

### Functional annotation and protein clustering

Some 11,837,198 protein-coding genes were identified from the 189,680 MGVs using Prodigal and genes were annotated based on HMM searches against protein family databases: KEGG^55^, TIGRFAM^56^, Pfam-A^57^, VOGDB (http://vogdb.org) and the Earth’s Virome viral protein families database^23^. All searches were performed using the hmmsearch utility in the HMMER package v.3.1b2 (ref. ^75^) with default parameters. Each gene was annotated by each database according to its top scoring alignment with a bit-score ≥50, except for Pfam and TIGRFAM where trusted cut-offs were used. Antibiotic resistance genes were identified using three tools: (1) the Resistance Gene Identifier v.5.1.0 (ref. ^62^) using option ‘–low_quality’ with gene-specific bit-score thresholds, (2) the NCBI AMRFinder tool v.3.8.4 (ref. ^61^) using default options and (3) the Resfams database^60^ using hmmsearch with HMM-specific bit-score thresholds. DGRs were identified using the tool DGRscan^67^ with default options. All proteins were clustered at 30% AAI and 70% alignment coverage using MMseqs2 v.10.6d92c^58^.

### Reporting Summary

Further information on research design is available in the Nature Research Reporting Summary linked to this article.

## Supplementary information

Reporting Summary

Supplementary Tables Supplementary Tables 1–7.

## Data Availability

Access to the full catalogue of viral genomes, protein clusters, diversity-generating retroelements and CRISPR spacers is provided without restrictions at https://portal.nersc.gov/MGV. Any requests for further data should be directed to the corresponding authors.

## Code availability

Supporting code, including our viral detection pipeline, is provided at https://github.com/snayfach/MGV.
