# Supplementary material for: Metagenomic compendium of 189,680 DNA viruses from the human gut microbiome
Source: Nat Microbiol. 2021 Jun 24;6(7):960–70. doi: 10.1038/s41564-021-00928-6 (PMC8241571; doi:10.1038/s41564-021-00928-6)
Supplement: Supplementary file 1 — Reporting Summary [file 41564_2021_928_MOESM1_ESM.pdf]

nature research

Corresponding author(s): Stephen Nayfach and Nikos Kyrpides

Last updated by author(s): April 15, 2021

Reporting Summary

Nature Research wishes to improve the reproducibility of the work that we publish. This form provides structure for consistency and transparency in reporting. For further information on Nature Research policies, see Authors & Referees and the Editorial Policy Checklist.

Statistics

For all statistical analyses, confirm that the following items are present in the figure legend, table legend, main text, or Methods section.

n/a	Confirmed	
☐	☒	The exact sample size (n) for each experimental group/condition, given as a discrete number and unit of measurement
☐	☒	A statement on whether measurements were taken from distinct samples or whether the same sample was measured repeatedly
☐	☒	The statistical test(s) used AND whether they are one- or two-sided<br>*Only common tests should be described solely by name; describe more complex techniques in the Methods section.*
☐	☒	A description of all covariates tested
☐	☒	A description of any assumptions or corrections, such as tests of normality and adjustment for multiple comparisons
☐	☒	A full description of the statistical parameters including central tendency (e.g. means) or other basic estimates (e.g. regression coefficient) AND variation (e.g. standard deviation) or associated estimates of uncertainty (e.g. confidence intervals)
☐	☒	For null hypothesis testing, the test statistic (e.g. F, t, r) with confidence intervals, effect sizes, degrees of freedom and P value noted<br>*Give P values as exact values whenever suitable.*
☒	☐	For Bayesian analysis, information on the choice of priors and Markov chain Monte Carlo settings
☒	☐	For hierarchical and complex designs, identification of the appropriate level for tests and full reporting of outcomes
☐	☒	Estimates of effect sizes (e.g. Cohen's d, Pearson's r), indicating how they were calculated

Our web collection on statistics for biologists contains articles on many of the points above.

Software and code

Policy information about availability of computer code

Data collection	Prodigal v2.6.3, HMMER v3.1b2, VirFinder v1.1, DIAMOND v0.9.25.126, Barrnap v0.9-dev, blast+ v.2.9.0, Bowtie v2.3.2, MCL v14-137, FAMSA v1.2.5, trimAL v1.4, FastTree v2.1.9, iTOL, MUMmer4 v4.0.0beta2, CRT, PILER-CR, AMRFinder v3.8.4, Resistance Gene Identifier v.5.1.0, MMseqs2 v10.6d92c, PSI-BLAST, CheckV v0.7.0, viromeQC, DGRscan
Data analysis	See software listed above

For manuscripts utilizing custom algorithms or software that are central to the research but not yet described in published literature, software must be made available to editors/reviewers. We strongly encourage code deposition in a community repository (e.g. GitHub). See the Nature Research guidelines for submitting code & software for further information.

Data

Policy information about availability of data

All manuscripts must include a data availability statement. This statement should provide the following information, where applicable:

- Accession codes, unique identifiers, or web links for publicly available datasets
- A list of figures that have associated raw data
- A description of any restrictions on data availability

Access to the full dataset of viral genomes, protein clusters, diversity generating retroelements, and CRISPR spacers is provided without restrictions at https://portal.nersc.gov/MGV. Any requests for further data should be directed to the corresponding authors.

Field-specific reporting

Please select the one below that is the best fit for your research. If you are not sure, read the appropriate sections before making your selection.

☒ Life sciences ☐ Behavioural & social sciences ☐ Ecological, evolutionary & environmental sciences

For a reference copy of the document with all sections, see nature.com/documents/nr-reporting-summary-flat.pdf

Life sciences study design

All studies must disclose on these points even when the disclosure is negative.

Sample size	We used 18,271 assembled human gut metagenomes for 11,810 samples. These represent all available datasets from the gut microbiome with SRA accession codes at the time we started our project.
Data exclusions	We excluded datasets that were not from a human stool sample or where SRA accession codes could not be determined.
Replication	Not applicable to our study. All the datasets analyzed were publicly available, and therefore we did not generate any additional data for replication.
Randomization	Not applicable to our study. Any conditions of the samples (e.g. geographic location or disease state) were already determined before our study began since the datasets were publicly available.
Blinding	Not applicable to our study for the same reason given above.

Reporting for specific materials, systems and methods

We require information from authors about some types of materials, experimental systems and methods used in many studies. Here, indicate whether each material, system or method listed is relevant to your study. If you are not sure if a list item applies to your research, read the appropriate section before selecting a response.

Materials & experimental systems

n/a	Involved in the study	
☒	☐	Antibodies
☒	☐	Eukaryotic cell lines
☒	☐	Palaeontology
☒	☐	Animals and other organisms
☒	☐	Human research participants
☒	☐	Clinical data

Methods

n/a	Involved in the study	
☒	☐	ChIP-seq
☒	☐	Flow cytometry
☒	☐	MRI-based neuroimaging
